# Making Biomolecular Simulations Accessible in the Post-Nobel Prize Era

**DOI:** 10.1371/journal.pcbi.1003786

**Published:** 2014-08-14

**Authors:** Qiang Cui, Ruth Nussinov

**Affiliations:** 1 Department of Chemistry and Theoretical Chemistry Institute, University of Wisconsin-Madison, Madison, Wisconsin, United States of America; 2 Cancer and Inflammation Program, Leidos Biomedical Research, Inc., Frederick National Laboratory for Cancer Research, National Cancer Institute, Frederick, Maryland, United States of America; 3 Sackler Institute of Molecular Medicine, Department of Human Genetics and Molecular Medicine, Sackler School of Medicine, Tel Aviv University, Tel Aviv, Israel

In 2013, three pioneers of computational biophysics and structural biology, Martin Karplus, Arieh Warshel, and Michael Levitt, were awarded the Nobel Prize in Chemistry. Although the citation focused on their innovative efforts on integrating quantum mechanical and classical mechanical models to study reactive processes in proteins, the award has also been seen by many researchers in the biomolecular simulation field as recognizing the tremendous value of computations for the investigation of biomolecules in general. From the days when proteins were modeled at the picosecond timescale using a united atom representation [1], or even as coarse-grained beads [2], in vacuum, to modern simulations that approach the millisecond timescale for a fully solvated protein [3], the biomolecular simulation field has, indeed, come a long way. Much of the progress has been due to the efforts of the three laureates, their contemporaries, and many others (e.g., their students) who were inspired by their dream of understanding life by studying “the jiggling and wiggling of atoms” [4]. One could only admire the tremendous courage, imagination, and vision that drove these three scientists to start pursuing their dream in an era when theoretical and computational chemistry largely focused on understanding the interactions and reactivity of small molecules.

Just as the Nobel Prize in 1998 to John Pople and Walter Kohn highlighted both the impact and emerging challenges of quantum chemistry, the 2013 Chemistry Prize should also further inspire us to ponder about the future of computational biology. Clearly, developing methodologies that further enhance the quantitative accuracy and/or complexity of computational models are important and being actively pursued by many researchers. On the quantitative aspect, several community-wide blind tests on observables such as solvation free energies, binding affinities, and pK_a_ values are being held. Provided that the results are disseminated in a constructive manner, these blind tests are highly valuable for helping the community converge towards the most robust and efficient computational algorithms and protocols. On the other hand, it is valuable to bear in mind that in many (certainly not necessarily all) investigations, quantitative computations represent a means to validate the model rather than the ultimate goal, which ought to focus on revealing the physical and chemical principles that govern the biological problem at hand. In other words, understanding qualitative trends is equally important. Therefore, building models with different levels of complexity and identifying robust features relevant to the biological problem remains an important research strategy. After all, in many mechanistic studies, whether at the molecular or cellular scale, the ultimate goal is to establish a conceptual framework to guide the development of novel mechanistic hypotheses and to stimulate new experiments to evaluate them.

Another important issue worth emphasizing in this “Post-Nobel Prize era” concerns making high-quality biomolecular simulation protocols available to the bioscience community, especially to young researchers who have just entered the field and perhaps even researchers who are primarily experimentalists. Such efforts will be essential to further enhancing the impact of biomolecular simulations while maintaining a high level of integrity in the result. In this issue of *PLOS Computational Biology*, Woodcock and coworkers have made a major step in this direction by describing a set of web-based tutorials and tools for the simulation package Chemistry at HARvard Molecular Mechanics (CHARMM) [5]–[7]; the tools are fittingly and playfully referred to as “CHARMMing.” The web-based tools make it straightforward to set up complex biomolecular simulations, including reduction potential computation for proteins and molecular dynamics simulations using a coarse-grained model. For even an expert in biomolecular simulation, it is often cumbersome to set up a new simulation that requires the generation of force field parameters for cofactors; CHARMMing is helpful in this context by providing an easy access to several automated small molecule force field generation services (e.g., the ParamChem web-server, the MATCH toolkit).

Importantly, CHARMMing goes beyond simply facilitating the set-up of biomolecular simulations by including carefully designed lessons on topics that range from basic simulation tutorials to advanced protocols such as quantum mechanical (QM)/molecular mechanical (MM) calculations and enhanced sampling techniques. The graphic interface allows the “students,” who take those lessons, to understand and modify CHARMM input scripts as well as visualize simulation results. Therefore, CHARMMing is valuable not only as a research tool, but also an educational module that can easily be incorporated into curriculum at both the undergraduate and early graduate level. As a result, CHARMMing is complementary to another valuable web-based research tool, CHARMM-GUI [8], which features a number of sophisticated functionalities, such as setting up membrane simulations [9] and absolute ligand binding affinity calculations [10]. We hope that the set of CHARMMing papers will help stimulate additional efforts in bringing advanced simulations, good computational practices, and thorough analysis of simulation results to the broader biological research community. Although pushing the limit of computational research via method development is always essential, an equally important goal is, to paraphrase what Martin Karplus once stated [11], that experimental (structural) biologists, who know their systems better than anyone else, will make increasing use of molecular dynamics simulations for obtaining a deeper understanding of particular biological systems.

This Editorial was first published as a blog post on PLOS Biologue on July 25, 2014.

## References

[1] McCammon JA, Gelin BR, Karplus M (1977) Dynamics of folded proteins. Nature 267: 585–590. 301613 10.1038/267585a0

[2] Levitt M, Warshel A (1975) Computer simulation of protein folding. Nature 253: 694–698. 1167625 10.1038/253694a0

[3] Shaw DE, Maragakis P, Lindorff-Larsen K, Piana S, Dror RO, et al (2010) Atomic-level characterization of the structural dynamics of proteins. Science 330: 341–346. 20947758 10.1126/science.1187409

[4] Feynman RP, Leighton RB, Sands M (1963) The Feynman Lectures in Physics. Reading: Addison-Wesley.

[5] Miller BT, Singh RP, Schalk V, Pevzner Y, Sun JJ, et al (2014) Web based computational chemistry education with CHARMMing I: Lessons and Tutorial. PLoS Comput Biol 10: e1003719. 25057988 10.1371/journal.pcbi.1003719 PMC4109840

[6] Pickard FC IV, Miller BT, Schalk V, Lerner MG, Woodcock HL III, et al (2014) Web based computational chemistry education with CHARMMing II: Coarse Grained Protein Folding. PLoS Comput Biol 10: e1003738. 25058338 10.1371/journal.pcbi.1003738 PMC4109841

[7] Perrin BS Jr, Miller BT, Schalk V, Woodcock HL III, Brooks BR, Ichiye T (2014) Web-based computational chemistry lessons in CHARMMing III: Reduction potentials of electron transfer proteins. PLoS Comput Biol 10: e1003739. 25058418 10.1371/journal.pcbi.1003739 PMC4110074

[8] Jo S, Kim T, Iyer VG, Im W (2008) CHARMM-GUI: A web-based graphical user interface for CHARMM. J Comp Chem 29: 1859–1865. 18351591 10.1002/jcc.20945

[9] Jo S, Lim JB, Klauda JB, Im W (2009) CHARMM-GUI Membrane Builder for Mixed Bilayers and Its Application to Yeast Membranes. Biophys J 97: 50–58. 19580743 10.1016/j.bpj.2009.04.013 PMC2711372

[10] Jo S, Jiang W, Lee HS, Roux B, Im W (2013) CHARMM-GUI Ligand Binder for Absolute Binding Free Energy Calculations and Its Application. J Chem Info Model 53: 267–277. 10.1021/ci300505n PMC3557591 23205773

[11] Karplus M, Kuriyan J (2005) Molecular dynamics and protein function. Proc Natl Acad Sci U S A 102: 6679–6685. 15870208 10.1073/pnas.0408930102 PMC1100762

